# Reliability of gradient-based segmentation for measuring metabolic parameters influenced by uptake time on 18F-PSMA-1007 PET/CT for prostate cancer

**DOI:** 10.3389/fonc.2022.897700

**Published:** 2022-09-29

**Authors:** Yu Ching Lau, Sirong Chen, Chi Lai Ho, Jing Cai

**Affiliations:** ^1^ Department of Health Technology and Informatics, The Hong Kong Polytechnic University, Hong Kong, Hong Kong SAR, China; ^2^ Department of Nuclear Medicine and Positron Emission Tomography, Hong Kong Sanatorium and Hospital, Hong Kong, Hong Kong SAR, China

**Keywords:** prostate cancer, PSMA, PET, uptake time, tumour delineation, tumour volume, metabolic tumour burden, tumour

## Abstract

**Purpose:**

To determine an optimal setting for functional contouring and quantification of prostate cancer lesions with minimal variation by evaluating metabolic parameters on 18F-PSMA-1007 PET/CT measured by threshold-based and gradient-based methods under the influence of varying uptake time.

**Methods and materials:**

Dual time point PET/CT was chosen to mimic varying uptake time in clinical setting. Positive lesions of patients who presented with newly diagnosed disease or biochemical recurrence after total prostatectomy were reviewed retrospectively. Gradient-based and threshold-based tools at 40%, 50% and 60% of lesion SUVmax (MIM 6.9) were used to create contours on PET. Contouring was considered completed if the target lesion, with its hottest voxel, was delineated from background tissues and nearby lesions under criteria specific to their operations. The changes in functional tumour volume (FTV) and metabolic tumour burden (MTB, defined as the product of SUVmean and FTV) were analysed. Lesion uptake patterns (increase/decrease/stable) were determined by the percentage change in tumour SUVmax at ±10% limit.

**Results:**

A total of 275 lesions (135 intra-prostatic lesions, 65 lymph nodes, 45 bone lesions and 30 soft tissue lesions in pelvic region) in 68 patients were included. Mean uptake time of early and delayed imaging were 94 and 144 minutes respectively. Threshold-based method using 40% to 60% delineated only 85 (31%), 110 (40%) and 137 (50%) of lesions which all were contoured by gradient-based method. Although the overall percentage change using threshold at 50% was the smallest among other threshold levels in FTV measurement, it was still larger than gradient-based method (median: 50%=-7.6% vs gradient=0%). The overall percentage increase in MTB of gradient-based method (median: 6.3%) was compatible with the increase in tumour SUVmax. Only a small proportion of intra-prostatic lesions (<2%), LN (<4%), bone lesions (0%) and soft tissue lesions (<4%) demonstrated decrease uptake patterns.

**Conclusions:**

With a high completion rate, gradient-based method is reliable for prostate cancer lesion contouring on 18F-PSMA-1007 PET/CT. Under the influence of varying uptake time, it has smaller variation than threshold-based method for measuring volumetric parameters. Therefore, gradient-based method is recommended for tumour delineation and quantification on 18F-PSMA-1007 PET/CT.

## Introduction

The clinical use of prostate specific membrane antigen (PSMA) ligand for PET imaging has revolutionized the diagnostic and therapeutic paradigm of prostate cancer. The usefulness of PSMA PET/computed tomography (CT) in guiding radiotherapy (RT) has been widely reported, especially for the detection of local recurrence and metastasis including lymph nodes (LN) and osseous lesions in which conventional imaging modalities may be less sensitive to depict (1, 2). It is clinically useful for the delineation of intraprostatic lesions without clear margin in primary disease and the localisation of oligometastatic lesions usually having small physical size at early biochemical recurrence after prostatectomy. Image-derived metabolic parameters such as tumour maximum standardized uptake value (SUVmax), functional tumour volume (FTV) and metabolic tumour burden (MTB) were reported to be useful for disease quantification. Contours on PSMA PET/CT was also shown to be of clinical significance in tumour volume delineation which led to changes in treatment plan (3–7).

Lesion contouring is labour-intensive in treatment planning workflow especially when contours are drawn manually. Threshold-based and gradient-based methods are clinically available semi-automatic segmentation on PET images with higher reliability and smaller inter-observer bias (8–10). Although different approaches and settings have been suggested for PSMA PET/CT, they are specific to clinical application and target lesion type which limits their practicality in routine practice (11–16).

PET tracer uptake is a pharmacokinetic process. The rates of tracer uptake in different tissues vary with tracer concentration and cellular microenvironment. Acquiring PET images at different time points gives rise to variations in metabolic parameters. Standardization of PET imaging protocol has been proposed (17, 18). Nevertheless, there is no consensus on the optimal uptake time for 18F-PSMA PET/CT as evident by the wide range of uptake time in previous studies (19–21). This technical factor varies from patient to patient, from scan to scan, even within a single imaging centre. However, there is no study evaluated the variations in metabolic parameters measured by gradient-based and threshold-based methods at different uptake times in clinical 18F-PSMA PET/CT examinations.

Therefore, an efficient and reliable lesion contouring method with smaller level of variation is of interest to clinical practice for this increasingly common and important image guidance. This study aimed to determine an optimal setting for contouring prostate cancer lesions on 18F-PSMA-1007 PET/CT with minimal variation under the influence of uptake time.

## Methods and materials

### Patient data

In this study, dual time point imaging was selected to mimic the varying uptake time in clinical setting. Clinical 18F-PSMA-1007 PET/CT examinations performed in the Hong Kong Sanatorium and Hospital from November 2019 to May 2021 were reviewed retrospectively under an Internal Review Board (IRB)-approved protocol. To allow direct comparison, only cases scanned using cross calibrated PET/CT scanners of the same model (Siemens Biograph Vision 600) were retrieved from image archive.

To obtain an appropriate representation of lesions requiring functional contouring for metabolic quantification in daily clinical practice, only patients who were [a] newly diagnosed by confirmed biopsy of primary disease and positive MRI findings without any medical treatments, or [b] presented with the first biochemical recurrence after prostatectomy without any post-operative treatments were selected. Considered that 18F-PSMA-1007 uptake can be non-specific, only positive lesions reported by certified nuclear medicine physician were included.

### Scan preparation and image acquisition

Pre-examination fasting was not required. Injected activity of 18F-PSMA-1007 was calculated according to patient’s weight (6.5-11.0 mCi). After intravenous injection, patients were requested to take a rest in preparation room for a minimum uptake period of 90 minutes before scanning. Whole-body scans were acquired after urination, spanning from base of skull to upper thigh. Patients performed normal breathing with both arms positioned above head. Non-contrast CT was performed (120 kVp, 90 mAs, pitch 0.8 and rotation time 0.5 second) followed by PET acquisition (static bed, 2 minutes per bed). Regional delayed imaging was performed as per physician’s order when clinically indicated. PET images were reconstructed using parameters optimized for small lesion depiction (4 iterations 4 subsets, gaussian filter 5 mm at FWHM, voxel size 1.65 x 1.65 x 1.5 mm, point spread function and time-of-flight options enabled).

### Image measurement

Images were loaded to imaging workstation (MIM 6.9, MIM Software INC., US). Both gradient-based and threshold-based tools used for contouring have been described earlier as follows (22). Gradient-based method is a textual analysis detecting the point having the greatest slope of lesion activity profile by calculating its spatial derivative. A starting point is defined near the centre of lesion. Six axes with visualized length are dragged out by user. The spatial gradients along these axes are calculated interactively. These axes are restricted to a large spatial gradient that detected near the edge of the lesion. The ellipsoid volume formed is used as a starting boundary for gradient detection. When mouse button is released, the lesion contour is created at the edge detected using the maximal spatial gradient along each axis. Threshold-based method creates contour in a user defined spherical volume covering the entire target lesion by including those having values larger than the chosen threshold in the form of percentage of lesion SUVmax.

Gradient-based and threshold-based methods using 40%, 50% and 60% of lesion SUVmax were used to create tumour contours on both early and delayed images. Tumour SUVmax, FTV and MTB (defined as the product of mean standardized uptake value (SUVmean) and FTV) were exported for analyses. All lesions were categorized into four groups according to their types: intra-prostatic lesions, LN, bone lesions, and soft tissue lesions in pelvic region. The uptake times of scans (from tracer injection and the start of acquisitions) were recorded.

### Data analysis

Contouring was considered completed if [a] the target lesion was delineated from background tissues and nearby lesions without the need of manual adjustment or smoothing, [b] the contour encompassed the hottest voxel of the target lesion, [c] in gradient-based method, none of the axes starting from the lesion centre has extended out of the lesion without detecting and restricted by a large spatial gradient near the edge, and [d] in threshold-based method, the contour was created without being constrained by the user defined spherical volume. Completion rate was determined for each segmentation method as:


$$\text{Completion Rate}=\;\frac{\text{Number of lesion with completed contouring}}{\text{Total number of lesion}}\;\times 100\%$$

To determine the levels of variation of segmentation methods, percentage changes in FTV and MTB of each completed contour pair were calculated as follows. A segmentation method that gives consistent FTV with percentage change approaching zero, is preferred.


$$\text{\% change in FTV}=\frac{{\text{FTV}}_{\text{delayed}}-{\text{FTV}}_{\text{early}}\;}{{\text{FTV}}_{\text{early}}}\times 100\%$$

$$\text{\% change in MTB}=\frac{\text{MT}{\text{B}}_{\text{delayed}}-\text{MT}{\text{B}}_{\text{early}}}{\text{MT}{\text{B}}_{\text{early}}}\times 100\%$$

Percentage change in tumour SUVmax of each lesion was calculated as:


$$\text{\% change in tumour SUVmax}=\frac{{\text{SUVmax}}_{\text{delayed}}-{\text{SUVmax}}_{\text{early}}\;}{{\text{SUVmax}}_{\text{early}}}\times 100\%$$

Lesion uptake pattern was determined by the percentage change in tumour SUVmax. Limits were adopted from a previous study on 68Ga-PSMA PET/CT as follows (23):

- “increase” for +10% or more, or
- “decrease” for -10% or more, or
- “stable” within ±10%.

Statistical analyses were performed using IBM SPSS Statistics (version 26, IBM Corp., US). Tumour SUVmax, FTV and MTB were compared between time points using paired t-tests. Percentage changes in SUVmax of lesions in newly diagnosed and post-prostatectomy group were compared using independent sample t-test. Two-sided p-value<0.05 was considered statistically significant.

## Results

A total of 275 lesions (135 intra-prostatic lesions, 65 LN, 45 bone lesions and 30 soft tissue lesions in pelvic region) in 68 patients (44 newly diagnosed and 24 with prostatectomy done) were included in this study. Same number of lesions was detected on both time points. Mean uptake time of early and delayed imaging were 94 ± 16.8 and 144 ± 14.3 minutes respectively.

### Completion rate

**Table 1** summarises the completion rates in all lesions and each lesion type. Contouring of all the 275 lesions were completed using gradient-based method. Completion rates were lower using threshold-based method. For each threshold level, the lowest rate was observed for intra-prostatic lesions because of failure to separate target lesions from nearby more intense lesions; the highest rate was seen in bone lesions which were usually having a well-defined margin. In all lesion types, the completion rates dropped with threshold level because of the inclusion of background activity when lesion contrast was insufficient.

**Table 1:** Completion rates of gradient-based and threshold-based segmentation methods.

	All lesions	Intra-prostatic lesions	LN	Bone lesions	Soft tissue lesions
Gradient	100%	100%	100%	100%	100%
Threshold-40%	31%	11%	51%	67%	23%
Threshold-50%	40%	19%	63%	76%	33%
Threshold-60%	50%	29%	71%	87%	43%

### FTV

The median percentage changes in FTV of all lesions and within each lesion type were summarised in **Table 2**. Gradient-based method outperformed threshold-based method at 50% (-7.6%) even though it gave the most consistent measurements among different threshold levels. In per lesion type analysis, gradient-based method generally demonstrated a higher consistency for all lesion types (-2.6% to 0%), without any significant difference detected between time points. On the contrary, FTV measured by threshold-based method were generally smaller on delayed time point, with relatively large decrease for LN and soft tissue lesions.

**Table 2:** Median percentage changes in FTV of gradient-based and threshold-based segmentation methods.

%	All lesions	Intra-prostatic lesions	LN	Bone lesions	Soft tissue lesions
Gradient	0.0	0.0	-2.0	-2.6	0.0
Threshold-40%	-8.8*	-5.9	-16.0*	-7.3*	-17.5
Threshold-50%	-7.6*	-4.4	-17.6*	-5.2	-13.7*
Threshold-60%	-12.0*	-4.5	-18.9*	-6.3	-15.2

(* = significant difference between time points [p<0.05]).

### MTB

MTB using gradient-based method generally demonstrated increases in all lesion types, ranged from 2.1% to 9.8%. Meanwhile, the changes using threshold-based method were diverse, ranged from -3.2% to 5.6% for threshold-40%, -8.1% to 7.4% for threshold-50% and -9.2% to 6.0% for threshold-60%. The increase observed for gradient-based method was compatible with the increase in tumour SUVmax.

### Tumour SUVmax

**Table 3** summarises the mean tumour SUVmax on early and delayed images. Increase in delayed SUVmax was observed [all p<0.05]. Mean percentage changes in SUVmax for intra-prostatic lesions, LN, bone lesions and soft tissue lesions were 10.2%, 11.1%, 12.0% and 10.0% respectively.

**Table 3:** Mean tumour SUVmax ± SD on early and delayed images.

	Early	Delayed
All lesions	8.7 ± 13.1	9.7 ± 14.6
Intra-prostatic lesions	11.3 ± 17.1	12.5 ± 19.0
LN	5.6 ± 5.8	6.2 ± 6.3
Bone lesions	6.8 ± 4.6	7.6 ± 5.2
Soft tissue lesions	6.8 ± 7.7	7.7 ± 12.5

### Uptake pattern

Majority of the lesions showed stable or increase pattern on delayed images, except a small proportion of intra-prostatic lesions (<2%), LN (<4%), bone lesions (0%) and soft tissue lesions (<4%) which demonstrated decrease pattern. There was a larger proportion of bone lesions in the post-prostatectomy group showing increase pattern, with a significantly larger mean percentage change in SUVmax than the newly diagnosed group (17.0% vs 9.3%, [p<0.05]).

### Efficacy for contouring heterogeneous lesions

**Figure 1:**
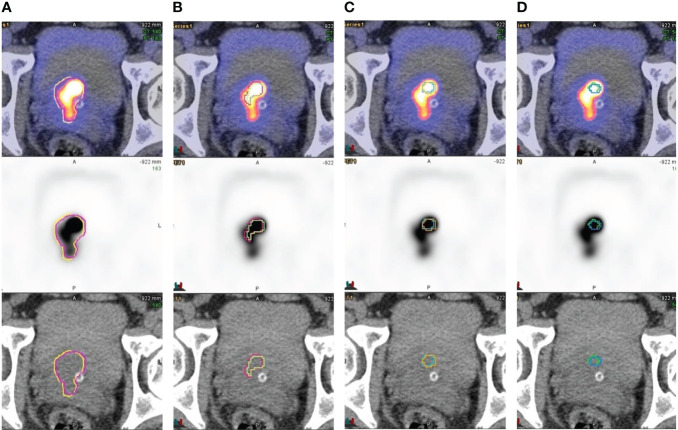
Contouring of a heterogenous prostatic mass involving bilateral base-mid gland TZ and R apex **(A)** gradient-based, **(B)** threshold-40%, **(C)** threshold-50% and **(D)** threshold-60%. Early and delayed contours were overlaid on axial plane. Threshold-based method failed to delineate the whole tumour volume using all that threshold values applied. Gradient-based contours delineated the tumorous activity with higher level of confidence.

**Figure 2:**
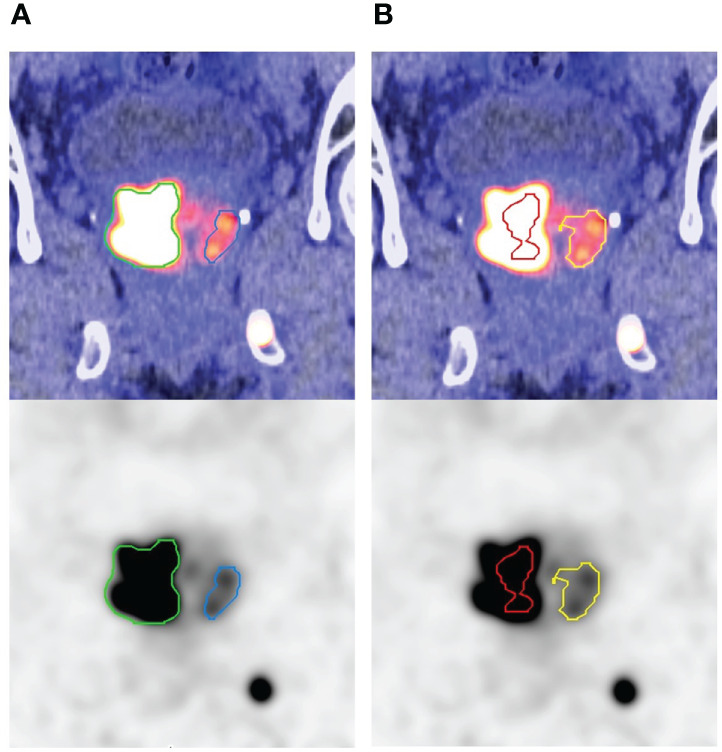
Countours on coronal plane of a heterogenous prostatic mass involving bilateral prostatic lobes by **(A)** gradient-based and **(B)** threshold-based methods (right lobe: 40% in red; left lobe: 60% in yellow). Different threshold levels were applied because a single level could not delineate tumours in right and left prostate lobes, which resulted in large discrepancy in disease extent of contours. Gradient-based contours improved the delineation with higher level of confidence.

Threshold-based method showed inadequacy for delineating heterogeneous lesions. **Figure 1** shows an example of gradient-based and threshold-based contours of a prostatic mass (early SUVmax 98). The threshold-based contours failed to include entire active sub-volume of the tumour no matter which threshold level was used, with a substantially smaller FTV and MTB when compared to the gradient-based contours. **Figure 2** shows another illustration of gradient-based and threshold-based contours of lesions in bilateral prostatic lobes. Tumour in left prostate could not be delineated even though threshold level at 40% was applied to whole prostate. When contoured separately using different threshold levels, the extent of tumorous activity being delineated on right lobe using 40% was significantly lesser than left lobe using 60% and those on gradient-based contours. Gradient-based method demonstrated a higher level of confidence for delineating the entire tumour volume than threshold-based contours.

## Discussion

The delineation of tumorous activity is a critical step during the translation of functional information on PET images to radiotherapy platform. The variations in metabolic parameters must be handled when treatment planning and disease monitoring are concerned. Fixing the exact uptake time is usually not feasible in clinical practice due to case scheduling, throughput and patients’ conditions. Therefore, addressing the variations from imaging work and measurement methodology can be another practical approach. Our results suggested that gradient-based method is more robust for tumour delineation and quantification on 18F-PSMA-1007 PET/CT. Having similar properties on targeting membrane glycoprotein overexpressed on prostate cancer cell surface with comparable detection sensitivity, common PSMA agents should exhibit similar image characteristics within their optimal uptake periods in which the working principles of the contouring algorithms are based on. Hence, our conclusions should be generalizable to other PSMA agents.

Although CT-based volume has been considered as standard of reference, contouring of prostate cancer lesions on CT images can be difficult and operator-dependent especially for lesions with ill-defined boundary and small size. Functional contouring using semi-automatic segmentation on PET images is an alternative. In our study, gradient-based method created contours for all lesions. Intra-prostatic lesions were the most difficult for threshold-based contouring. The lower completion rate can be attributed to the presence of nearby hot lesions and the lower lesion contrast. Although a higher threshold value can be useful, it results in shrinkage of tumour contour which may be unrealistic for gross tumour volume. In contrast, gradient-based method relies on the rate of change of neighbouring voxel values for edge detection, which is more versatile under challenging conditions. Tumour heterogeneity can be another issue for generating contours. **Figure 1** shows an example of another problem in contouring of a heterogeneous intra-prostatic lesion using threshold-based method. The maximum voxel value is much higher than other active sub-volumes within the tumour. Threshold-based method failed to delineate the whole tumour volume using all the threshold values applied. The FTV and MTB were considerably smaller when compared to gradient-based method. Moreover, adjusting threshold level on lesions showing different tracer avidity of the same patient is sometimes necessary when they cannot be contoured using a single level, which subsequently requires manual correction because of a large discrepancy on disease extent in contours as illustrated in **Figure 2**. Gradient-based method has improved the delineation of heterogeneous tumours in both situations, which echoes with the findings from a recent study (8). Therefore, gradient-based method is more robust than threshold-based method in clinical setting.

SUVmax is known to vary with uptake time. Despite, the degree of variation as observed in this study (**Table 3**) may be less significant in clinical context when coexisted with inter-patient and intra-patient variability in PSMA biodistribution (24, 25). Notwithstanding, the repeatability of PSMA PET/CT was proven and shown to be similar to 18FDG PET/CT that has been extensively used clinically with confidence (26, 27). The effect of lesion contouring using common segmentation methods on volumetric parameters with such variation in SUVmax and image appearance is the main concern of this study. Gradient-based and threshold-based segmentation methods have been evaluated in various aspects including the accuracy for delineating true tumour volume verified by histopathological evidence, the accuracy of spatial measurement using phantom as well as the inter-operator difference. In fact, a threshold value between 40% and 60% is commonly adopted for PSMA PET/CT threshold-based lesion segmentation in RT planning (14, 28–31). A recent study reported that FTV measured by threshold-based method using 55% had the highest correlation to CT volume among other values for metastatic lymph nodes (16). Although our findings revealed that threshold value at 50% gives relatively stable FTV and MTB measurements, the use of threshold-based method becomes questionable under the variation in tumour volume that we observed (corresponding to the ~16-19% decrease in FTV of LN for threshold-based using 50-60% in **Table 2**). In addition, dynamic uptake patterns of oligometastasis on 68Ga-PSMA PET/CT was reported by a previous study showing a larger proportion (21%) of bone lesion with decreased uptake on delayed time point (23). This further suggests the limitation of using threshold-based method on 68Ga-PSMA PSMA PET/CT as it relies on direct computation on SUVmax. Furthermore, the accuracy of gradient-based method for volumetric measurement at high lesion contrast commonly seen on PSMA PET/CT has been validated recently (32). In this regard, gradient-based method is a better option with smaller variation.

Gradient-based algorithm can benefit the current practice in different ways, which relies heavily on manual drawing and threshold-based method. Gradient-based contour is 3-dimensional which has a high degree of operator independence. The uncertainty of margin delineation is relatively low when compared to simple numerical analysis. The reasonable computing time also enables handy and efficient contouring for clinical use. Nevertheless, there are some precautions when operating the tool on images. Firstly, it is difficult to select the same seed in active volume by eyeballing which may affect the analysis of the tumour activity profile and result in slightly different contours for repeating attempts. Secondly, single attempt may not be able to include the entire volume of a heterogeneous lesion, especially when the slope of activity gradient is not constant along its margin. Repeated drawing is necessary to append tumour volume that is missing on the original contour. Since the operation requires a certain level of human input and experience, user training is essential to standardize the practice within a workgroup. It is also important that contouring of low count lesion using gradient-based method is subject to a larger uncertainty because textual analysis is primarily affected by high image noise and low lesion contrast. Nonetheless, its performance is still better than threshold-based method under these challenging conditions.

MTB is an image-based quantification marker of tumour burden in oncology. It is often used for monitoring systemic therapy in prostate cancer. In PET image quantification, increase in SUVmax is usually associated with increase in SUVmean. Recalling the mathematical definition of MTB (the product of SUVmean and FTV), it is plausible that an increased tumour SUVmax together with a stable FTV will result in an increased MTB. Compared to the decrease in MTB of LN and soft tissue lesions using threshold-based method, the increase using gradient-based method can be explained. Therefore, gradient-based method should be more reliable than threshold-based method for the measurement of MTB. It is noteworthy that measurement error propagates by the multiplication of two factors with individual variability. Minimizing variations in these parameters would become more critical for clinical applications using MTB which should be specific and confined by the measurement methodology.

There are limitations in this study. Physiologic motions such as bowel movement and urine accumulation in bladder are inevitable, even if patients are requested to stay on scanner between the acquisitions. During the examinations, patients were repositioned for delayed imaging. The displacement of internal body structures and body positioning may deform soft lesions, causing inherent variation which is not related to uptake time. However, this limitation is also present in real clinical situations between simulation and subsequent treatments. It is also noteworthy that benign lesions could not be completely excluded from our samples without histopathological or longitudinal evidence. In view of this, the inclusion or exclusion of lesions was not purely determined by their tracer avidity. The clinical reporting performed by nuclear medicine physicians often took other clinical factors and concomitant image findings into consideration, such as CT appearance, overall disease extent and patient’s clinical history. Nevertheless, the true metabolic nature of lesion should have a relatively small impact on our findings about the consistency of functional contouring methods under the variation of PET imaging.

## Conclusion

With a high completion rate, gradient-based method is reliable for prostate cancer lesion contouring on 18F-PSMA-1007 PET/CT. Under the influence of varying uptake time, it has smaller variation than threshold-based method for measuring volumetric parameters. Therefore, gradient-based method is recommended for tumour delineation and quantification on 18F-PSMA-1007 PET/CT.

## Data availability statement

The raw data supporting the conclusions of this article will be made available by the authors, without undue reservation.

## Ethics statement

The studies involving human participants were reviewed and approved by Institutional Review Board of the Hong Kong Polytechnic University. Written informed consent for participation was not required for this study in accordance with the national legislation and the institutional requirements.

## Author contributions

YCL, SC, CLH, and JC contributed to study design, methodology development, results interpretation, and manuscript review. CLH offered administrative and material support for clinical data and imaging data collection. YCL wrote the manuscript. JC supervised the study. All authors contributed to the article and approved the submitted version.

## Funding

This research was partly supported by research grants of Project of Strategic Importance Fund (P0035421) from The Hong Kong Polytechnic University.

## Conflict of interest

The authors declare that the research was conducted in the absence of any commercial or financial relationships that could be construed as a potential conflict of interest.

## Publisher’s note

All claims expressed in this article are solely those of the authors and do not necessarily represent those of their affiliated organizations, or those of the publisher, the editors and the reviewers. Any product that may be evaluated in this article, or claim that may be made by its manufacturer, is not guaranteed or endorsed by the publisher.
